# Evaluation of an innovative mHealth-based integrated modality for smoking cessation in Chinese smokers: protocol for a randomized controlled trial

**DOI:** 10.1186/s12889-023-15448-7

**Published:** 2023-03-25

**Authors:** Shuilian Chu, Lin Feng, Yingting Zuo, Hang Jing, Di Zhang, Zhaohui Tong, Ju Shi, Haomiao Ma, Zhijin Zhang, Lirong Liang

**Affiliations:** 1 Department of Research on Tobacco Dependence Therapies, Beijing Institute of Respiratory Medicine and Beijing Chao-Yang Hospital, Capital Medical University, No.8 Gong-Ti-Nan-Lu, Chaoyang District, Beijing, 100020 China; 2 Department of Respiratory and Critical Care Medicine, Beijing Institute of Respiratory Medicine and Beijing Chao-Yang Hospital, Capital Medical University, Beijing, China; 3 School of Economics, Peking University, Beijing, China

**Keywords:** Mobile health, Smoking cessation, WeChat app, Integrated modality, Randomized controlled trial

## Abstract

**Background and aims:**

Developing accessible, affordable, and effective approaches to smoking cessation is crucial for tobacco control. Mobile health (mHealth) based interventions have the potential to aid smokers in quitting, and integrating treatments from multiple sources may further enhance their accessibility and effectiveness. As part of our efforts in smoking cessation, we developed a novel behavioral intervention delivery modality for smoking cessation that integrated three interventions using the WeChat app, called the “Way to Quit” modality (WQ modality). It is presented here the protocol for a randomized controlled trial evaluating the effectiveness, feasibility, and cost-effectiveness of the WQ modality in Chinese smokers.

**Methods:**

Eligible participants (*n* = 460) will be recruited via online advertisement in Beijing, China. They will be randomly assigned to receive either quitline-based treatment (QT, *n* = 230) or WQ modality-based treatment (WQ, *n* = 230) using a block randomization method. Participants in the QT group will receive telephone-assisted treatment over a four-week period (multi-call quitline protocol), while those in the WQ group will receive integrated interventions based on the WQ modality for four weeks. A four-week supply of nicotine replacement therapy (gums) will be provided to all participants. Participants will be asked to complete phone or online follow-up at 1, 3, 6, and 12-months. At 1-month follow-up, individuals with self-reported smoking abstinence for more than 7 days will be invited to receive an exhaled carbon monoxide (CO) test for biochemical validation. The primary aim is to determine whether the WQ modality is effective in assisting smokers in quitting smoking. The secondary aims are to evaluate the acceptability, satisfaction, and cost-effectiveness of the WQ modality.

**Discussion:**

If the WQ modality is determined to be effective, acceptable, and affordable, it will be relatively easy to reach and provide professional cessation treatments to the communities, thus helping to reduce the disparities in smoking cessation services between different regions and socioeconomic groups.

**Trial registration:**

Chinese Clinical Trial Registry: ChiCTR2200066427, Registered December 5, 2022.

**Supplementary Information:**

The online version contains supplementary material available at 10.1186/s12889-023-15448-7.

## Background

Tobacco use is the leading cause of preventable morbidity and mortality in the world, and quitting smoking is known as the best approach to reduce these hazards [1]. Due to nicotine dependence, it is necessary to offer help to quit tobacco use [1]. Although many evidence-based treatment approaches to smoking cessation have been developed, the financing of smoking cessation services, such as tobacco quitlines, is a significant challenge for many governments, particularly those in low- and middle-income countries [2]. Consequently, many smokers do not have access to cessation services, and their quit rates are low [2].

This situation also exists in China, where more than 300 million Chinese people continue to use tobacco [3]. Among these smokers, 36.4% had tried to quit in the past 12 months, whereas more than 90% of them had never received any professional help [3]. A nationwide survey showed that there are only 366 smoking cessation clinics in mainland China [4], which leaded to smokers in most parts of China did not access to face-to-face treatment from smoking cessation specialists. Evidence has shown that the tobacco quitline is effective and could remove barriers that may hinder face-to-face service delivery [5]. However, there are only three quitlines in mainland China, all of which are facing the challenges of low awareness and utilization due to a lack of financial and policy support [6–8]. In addition, more and more people prefer to communicate via chat applications on their smartphones rather than by telephone [9]. Therefore, it needs to develop novel accessible, affordable, and effective approaches to assist smokers in quitting.

The rapid development of mobile health (mHealth) technology makes it possible to address the above concerns [10]. Moreover, during the COVID-19 pandemic, people preferred to obtain medical and health services through mHealth [11, 12], and this trend may continue in the post-epidemic era [13]. A variety of mHealth approaches, such as short message service (SMS) texting, web, social media, and mobile applications (apps), have existed to deliver smoking cessation behavioral interventions [10] and demonstrated the potential to assist smokers in quitting smoking [14, 15]. But at the same time, some limitations were also found that may reduce their availability and effectiveness, such as non-tailored contents, lack of interactivity, etc. Given the dynamic, quickly evolving nature of the technology, a possible strategy to overcome these limitations has been proposed that is to integrate interventions from multiple sources [10], but it still needs high-quality evidence to support it.

China is regarded as the fastest growing smartphone market, and has the largest smartphone user group (*n* > 850 million people) in the world [16]. WeChat is the most popular app in China, which has become a major tool for communication, entertainment, and payment for Chinese smartphone users [17]. In 2021, an innovative mHealth-based integrated modality for smoking cessation including three interventions using the WeChat app, called the “Way to Quit” modality (WQ modality), was developed in a large urban general hospital in Beijing, China. This hospital is the first hospital to set up a smoking cessation clinic and a free national quitline in mainland China, which makes it has outstanding clinical and research capability in smoking cessation services [7, 8]. A preliminary evaluation of the WQ modality conducted in 12 provinces and cities in western China indicated that it has been effective in assisting Chinese smokers in quitting [18].

This paper describes the protocol for a randomized controlled trial (RCT) that will evaluate the effectiveness, feasibility, and cost-effectiveness of the WQ modality in Chinese smokers. The primary aim of this study is to evaluate the effectiveness of the WQ modality in assisting smoking cessation. Secondary aims are: 1) to evaluate the acceptability and satisfaction of the WQ modality, and 2) to evaluate its cost-effectiveness. We hypothesize that the WQ modality would increase the smoking abstinence rate compared to quitline.

## Methods

### Study design

**Fig. 1:**
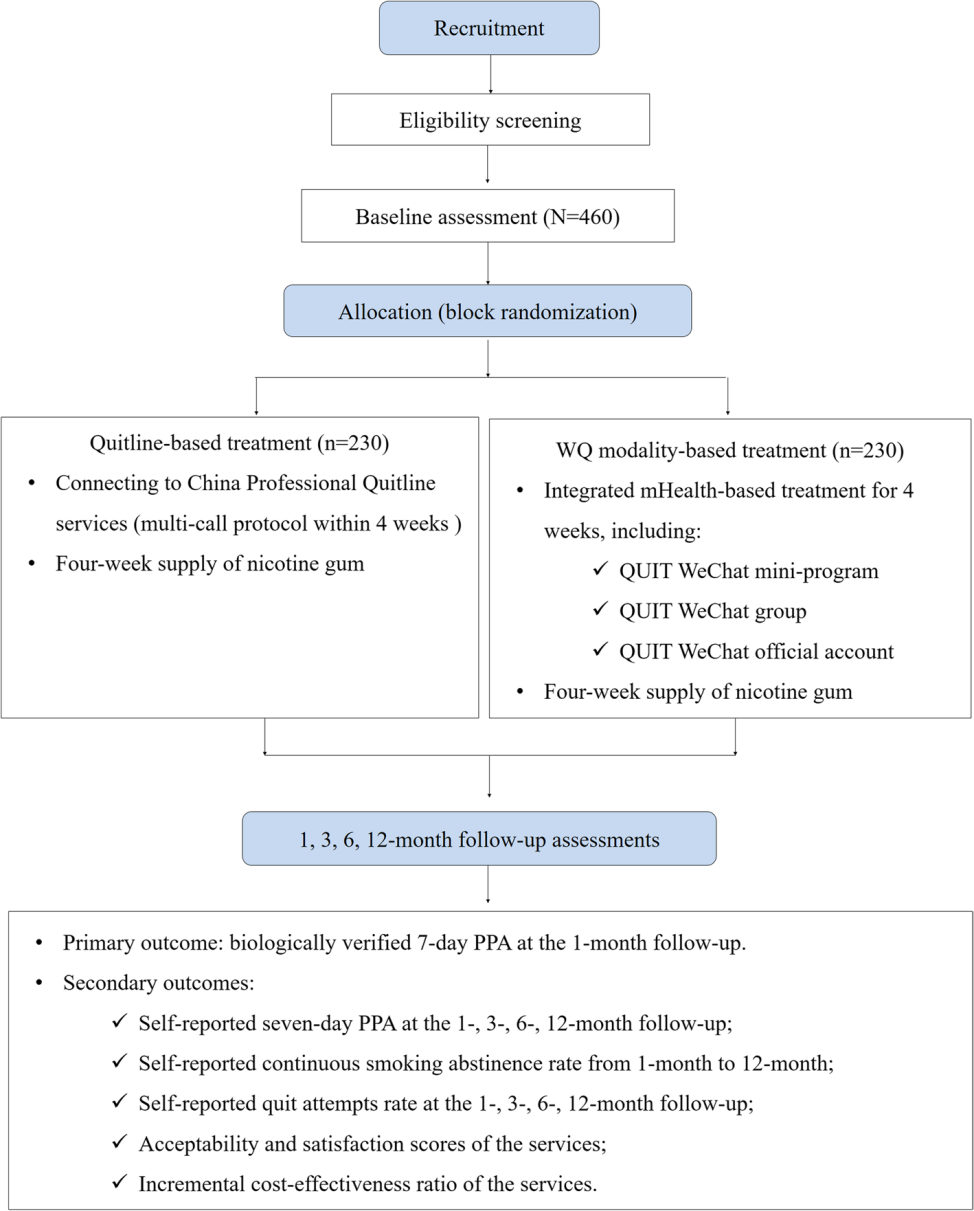
CONSORT diagram depicting trial design. Footnotes: WQ modality, “Way-to-Quit” modality; PPA, point prevalence of abstinence

This study will be a two-group, open label, randomized, parallel controlled trial among Chinese smokers in Beijing, China. Eligible participants will be randomized in a 1:1 ratio into the control group (quitline-based treatment, QT) or the intervention group (WQ modality-based treatment, WQ). All participants will complete baseline, and 1-, 3-, 6-, and 12-month follow-up assessments. The flow chart is shown in Fig. 1. This protocol has been designed in accordance with the Standardized Protocol Items: Recommendations for Interventional Trials (SPIRIT) guidelines and checklist (Additional file 1).

### Participants and recruitment

Participants will be Chinese smokers. The inclusion criteria are: 1) 18–65 years of age; 2) smoked at least 100 cigarettes in lifetime; 3) currently smoke at least 5 cigarettes per day; 4) willing to make a quit attempt within 1 month; 5) possess a smartphone and a WeChat account, and could operate WeChat app skillfully; 6) promise to complete follow-up on time. Exclusion criteria include: 1) difficulty in understanding the questionnaire; 2) acute exacerbation of chronic cardiopulmonary disease; 3) having received coronary surgical interventions (such as coronary angiography, percutaneous coronary interventions, etc.) in the last month or will receive these interventions in the next month; 4) discharge within 1 month; 5) medical conditions that preclude the use of nicotine replacement therapy (NRT); 6) currently using smoking cessation medications or other aids, including NRT, bupropion, varenicline, electronic cigarettes and other mHealth-based tools, such as web, SMS texting, apps, etc.; 7) being enrolled in another smoking cessation study.

Eligible participants will be recruited through online advertisement. Individuals who are interested in participating will screen a Quick Response (QR) cord to complete a screening questionnaire to ascertain their eligibility for the study. Eligible individuals will be provided an electronic consent (e-consent) form online. After reading the e-consent, they should click the agree button at the end of the form to indicate their consent to participate in the study. The participant recruitment has been started since December 15, 2022, and is ongoing.

### Randomization and allocation

Participants will be randomized in a 1:1 ratio to the QT group or the WQ group using a block randomization method with randomly selected block sizes (4 or 6). This study is an open-label trial. A non-participating staff member will generate a randomization sequence via SAS 9.4 software which will be concealed using sealed opaque envelopes.

### Interventions

#### Quitline-based treatment (QT group)

Participants in the QT group will receive standard proactive quitline services from China Professional Quitline (400–888-5531) for 4 weeks using a multi-call quitline protocol [19]. The first session will focus on quitting history, motivation, self-efficacy, social support, and planning in advance of the quitting date. Smokers will be reminded in the second session to quit smoking completely from the quitting date, be provided with the practical information on how to quit. The sessions followed the quitting date (up to three sessions) will focus on building cessation skills and relapse prevention. Three Chinese-speaking counselors will provide the quitline services. All of them possess at least a bachelor’s degree in medicine or nursing, and have received more than 60 h of training in smoking cessation counseling.

To improve the participants’ adherence of the quitline intervention protocol, the counselors will call them at three time points for three consecutive days during each session, arrange the next session time for the participants, and contact the participants’ relatives and friends whenever necessary.

#### WQ modality-based treatment (WQ group)

##### Conceptual framework of the WQ modality

**Fig. 2:**
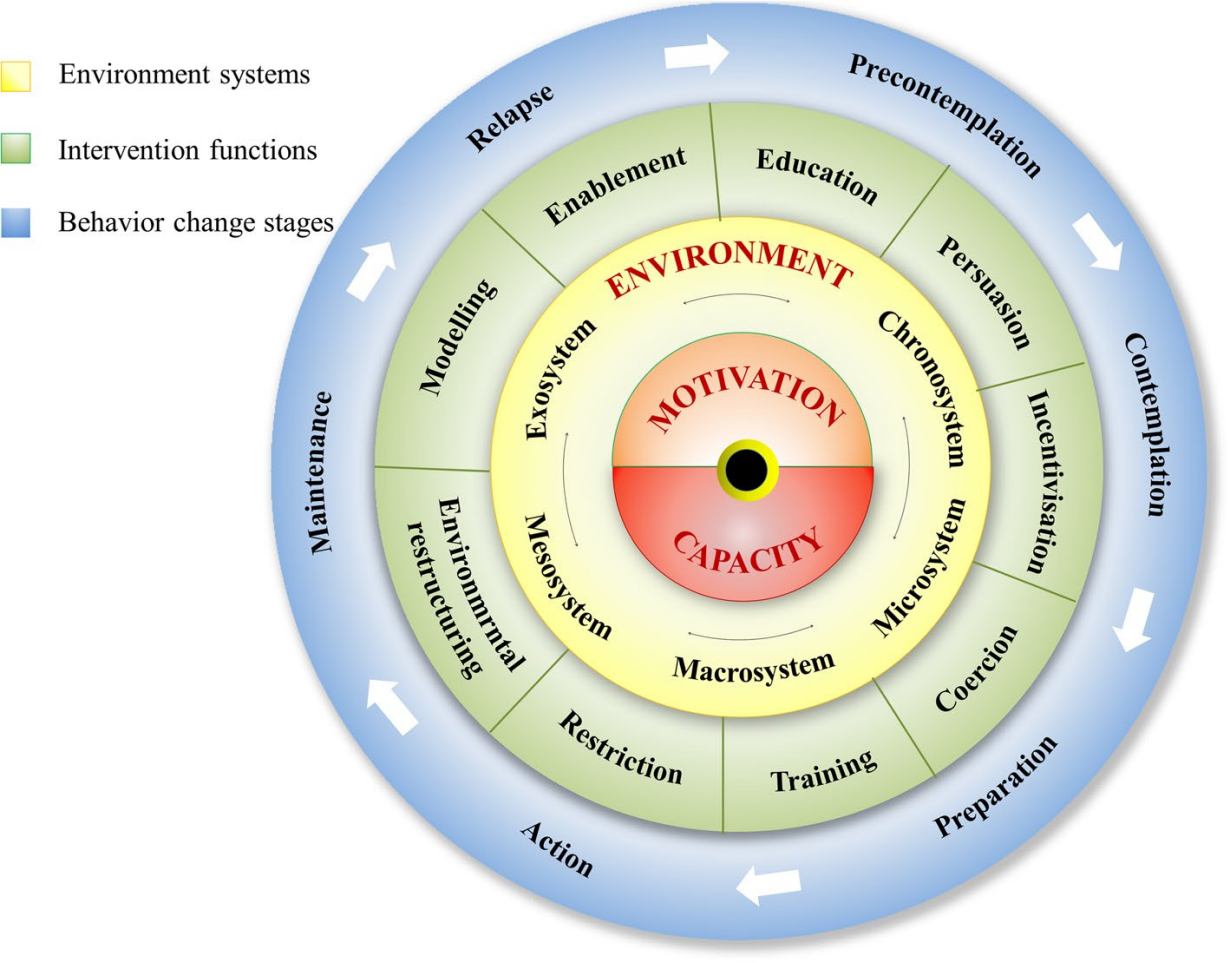
Conceptual framework of the “Way-to-Quit” modality

By integrating three behavior change theories, including the COM-B model (C-capability, O-opportunity, M-motivation, and B-behavior) [20], Ecological Systems Theory (EST) [21] and Transtheoretical Model (TTM) [22], we constructed the conceptual framework of the WQ modality, named Integrated COM-B/ Ecological systems/ Transtheoretical model (I-CET model), as shown in Fig. 2. In the I-CET model, three potential factors (Capability, Motivation, and Environment) could influence individual behaviors. Motivation is all those brain processes that energize and direct behavior, including habitual processes, emotional response, and analytical decision-making [23]. Capability is the individual’s psychological and physical capacity to engage in the activity concerned, such as having the necessary knowledge and skills [20]. Environment is defined as all the factors or resources that lie outside the individual that make behavior possible or prompt it (including microsystem, mesosystem, exosystem, macrosystem, and chronosystem) [21]. To promote the occurrence of behavioral change, nine intervention functions are determined based on the behavior change wheel (BCW) [20], as shown in Fig. 2. This is not a linear model in that components within the behavior system interact with each other as do the functions within the intervention layer. The definitions of these intervention functions have been described by Michie et al. [20]. In addition, the interventions need to match the stages of behavior change (including precontemplation, contemplation, preparation, action, maintenance, and relapse [22]), in order to provide tailored interventions.

##### WQ modality-based treatment

The WQ modality integrated three WeChat app-based cessation services, including the QUIT WeChat mini program (WMP), the QUIT WeChat group (WG), and the QUIT WeChat official account (WOA). Screenshots of these services have been shown in our previous study [18]. They are developed based on the I-CET model and connected with each other [18]. Since the target population is smokers who plan to quit in the next month, the WQ modality is mainly aimed at the stages of preparation, action, and maintenance, which are focus on motivating to quit, building skills for coping with smoking cravings, and preventing relapse, respectively.

The QUIT WMP is an application function module embedded in the WeChat app [24] (similar to cessation apps [25–27]), which could assist smokers in developing the capacity to quitting smoking and motivating them to do so. It was developed based on the behavior change techniques (BCTs) [28], and smoking cessation treatment guidelines [10, 29]. The detailed description of its functions is shown in Table 1. In addition, the QUIT WMP will automatically send stage-matched short messages, articles or videos via the WeChat app to the users once a day for 4 weeks from the quit date. A list of weekly topics and examples of messages is shown in Table 2. It can be accessed by scanning a QR code (Additional file 2) or searching Chinese ‘Jie Yan You Dao’ in the WeChat app without downloading or installing [24]. The QR code and introduction video of the QUIT WMP will be sent to the participants via the WeChat app after allocation. To increase the participants’ adherence of using the QUIT WMP, daily usage reminders messages will be sent in the WeChat group. Moreover, we will download the participants’ usage data of the QUIT WMP every week, and remind those who use the program less than 5 days per week by telephone.

**Table 1:** Detailed description of functions of the QUIT WeChat mini-program

Functions	Sub-functions	Description
Registration	Recording current smoking behavior	Record amount smoked, smoking duration, price of cigarettes, tar content of cigarettes, pattern of smoking behavior, etc
	Recording past history of quit attempts	Record number and duration of past quit attempts
	Testing the level of nicotine dependence	Provide a standardized test to help users assess their level of nicotine dependence
	Assessing quitting readiness	Assess motivation to quit and the stage of smoking cessation
Facilitating action plan	Setting a quit date	Help users to set a quit date
	Setting a plan for reducing smoking	Help users to set the daily maximum smoking consumption and the time between two cigarettes
	Listing reasons for quitting	Let users write down the reasons for quitting smoking and display it on the home page of the mini-program
	Resetting a quit plan	Encourage users to reset a quit date when they relapse
Recording quitting	Recording the duration of quitting	Prompt self-recording and displaying how many days the users not smoke
	Providing rewards on quitting	Give users a virtual medal of different levels according to the duration of quitting
	Sharing quitting achievements	Users could share their quitting achievements, including duration of quit smoking, the benefits from quitting, and the virtual medal obtained in the mini-program, to social media by an automatically generated picture
Benefits from quitting	Hazards reduced from quitting	Providing the information of reducing cigarettes and tar intake according to the number of quitting days and their previous daily cigarette consumption
	Cost saving from quitting	Providing the information of cost saved from not smoking according to the number of quitting days and their previous daily expenditure on smoking
	Health benefits from quitting	Providing the information of health benefits from not smoking according to the number of quitting days, such as the elimination of exhaled carbon monoxide
Coping with cravings	Recording smoking craving	Prompt self-recording the number of smoking cravings
	Motivational messages	Providing motivational messages to help users overcome cravings
	Coping toolkit	Providing skills to cope with cravings, including guiding relaxation and mindfulness exercises, challenging automatic thoughts, tips for coping with stress, etc
Reducing smoking	Calculator for daily smoking cigarettes	Calculating and displaying daily smoking cigarettes
Quitting diary	Recording smoking cues	Help users to record and identify smoking cues
	Advising on changing routine	Advise users to change daily routines to minimize exposure to smoking cues
Quitting tasks	Setting graded tasks	Prompt users to complete online assessment questionnaires at 1 week, 1 month, 2 months, 3 months, and 6 months after the quit date
	Assessing smoking cessation progress	Give feedback arising from assessment of current self-reported behaviours and progress towards becoming a permanent non-smoker
	Barrier identification and problem solving	Help users to identify the barriers (e.g. nicotine withdrawal symptoms, stress, etc.) that might make it harder to stay off cigarettes and provide personalized suggestions according to the barriers (e.g. how to deal with withdrawal symptoms, eliminate stress, and obtain social support, etc.)
	Strengthening ex-smoker identity	Encourage those who have quit smoking to construct a new identity as never smokers
	Preventing relapse	Provide users information about how relapses occur and to provide coping skills for preventing relapses
Medication reminder	Assessing medication(s) using experiences	Assess usage, side effects and benefits experienced of medication(s) that the smoker is currently using
	Setting a medication use plan	Set the daily time and amount of medication use
	Medication use reminder	Remind users to take medication on time
Additional supports	Quitline	Give information about tobacco quitline (400–888-5531), and develop the “One Click Call” function
	Smoking cessation clinic	Provide online appointment of smoking cessation clinic
	Online consultation	Provide the link of QUIT WeChat group to obtain real-time online consultation
	Online self-help materials	Offer the link to QUIT WeChat official account to obtain online self-help materials for smoking cessation
Users Information	Quitting information	Such as duration of quitting and the number of attempts to quit
	Motivation to quit	Reason for quitting smoking
	Achievements	Virtual medals and accumulate points

**Table 2:** The WQ^a^ modality-based treatment by week

Week	Stage	Topic	Example of messages automatically sent by the QUIT WMP^b^	Interventions in the QUIT WG^c^			
				**Group interventions**	**Topic discussion**	**Quit tasks**	**The QUIT WMP functions recommended to use**
Week 1	Preparation	Motivating to quit	Why do you want to quit smoking? Please write your reasons and put it where you can see it every day	Health hazards of smoking and benefits of quitting smoking	Why do I quit smoking?	Sending a screenshot of using the QUIT WMP in the online group every day	Reducing smoking Quitting diary
Week 2	Action	Setting a quit day	Please choose a date as the quit day, such as birthday, holiday, anniversary, etc	Facilitating a quit plan	Why did I fail to quit smoking in the past and how to deal with it this time?	Setting a quit day and announcing a quit declaration in the online group	Facilitating action plan Recording quitting Coping with cravings
Week 3	Maintenance	Coping with smoking craving	You may feel depressed after quitting smoking. Please do some things to make yourself happy, such as calling your best friends, watching movies, and shopping, etc	Skills to cope with smoking craving	How did I cope with smoking craving?	Sharing quitting achievements picture in the online group	Recording quitting Benefits from quitting Coping with cravings
Week 4	Maintenance	Preventing relapse	Refuse accidental smoking! The most difficult period is almost over	How to prevent relapse	What benefits do I get from quitting smoking?	Sharing smoking cessation experience in the online group	Recording quitting Benefits from quitting Quitting tasks

^a^WQ, Way to Quit

^b^WMP, WeChat mini-program

^c^WG, WeChat group

The QUIT WG is an online group chat function embedded in the WeChat app (up to 500 persons), which could offer real-time online cessation counseling, group interventions, and facilitate the formation of mutually reciprocated, strong, and long-lasting social bounds that support smoking cessation in a similar manner to that of Twitter and Facebook [30–32]. Three counselors are arranged to provide online counseling, and a group administrator is arranged to manage the group and encourage participant to send message. Stage-matched online group interventions will be provided via two manners, including video courses on smoking cessation by clinical expert, and Q&A session after the courses and at 12:00 a.m.-13:00 p.m. from Monday to Friday. We also conducted interactive activities, including topic discussion and quit tasks. Table 2 shows a list of weekly topics and examples of group interventions and interactive activities. Moreover, we introduce the "herd effect" model (opinion leader model) into the WQ modality. Herding is a phenomenon by which individuals follow the behavior of others [33]. It has been reported that people tend to emulate opinion leaders (leading sheep) who could influence others’ opinions, attitudes, beliefs, motivations, and behaviors of others [34], and thus can effectively change many behaviors [35–37].Ten self-selected opinion leaders from the participants will be enrolled as the “Quitting Pioneer” to set an example of successful quitter, share experience of smoking cessation, and promote other smokers to participate in interaction activities.

The QUIT WOA could provide electronic self-help materials for smoking cessation, which could be the material library of the WQ modality. The materials are classified according to the stage of smokers and updated weekly. In addition, the information of online group interventions and interactivities will be also uploaded in the QUIT WOA (named Smoking Cessation College) for smokers to download at any time.

#### Discontinuing or modifying allocated interventions

Participants who experience severe withdrawal symptoms (determined by smoking cessation specialist who does not participate in the study) during the intervention period will stop receiving allocated interventions and be referred to a smoking cessation clinic in a large general hospital in Beijing for intensive treatment.

#### Other interventions

All participants will receive a 4-week supply of NRT gum by mail (300 pieces). The gum is FDA-approved Nicorette brand 2 mg nicotine mint gum (McNeil Sweden AB, Inc., Helsingborg, Sweden). Participants will be directed to use the gum as needed when they encounter situations or cues that tempt them to smoke, or in anticipation of such situations [38]. A smoking cessation material with a video link to the instruction of medication use will be mailed with the gum.

After receiving the allocation interventions for 4 weeks, participants in the QT group will receive reactive smoking cessation counseling via telephone for one year, and participants in the WQ group will also receive the reactive counseling via the WeChat group. Smoking cessation interventions other than the allocated intervention are prohibited during the study, such as bupropion, varenicline, electronic cigarettes and other mHealth-based tools, such as web, SMS texting, apps, etc.

### Measures

#### Baseline measures

Baseline data will be collected by an online questionnaire using the Tencent Questionnaire platform, including demographic information, smoking history, previous quit attempts, and comorbidities, etc. Nicotine dependence is measured by the Fagerstrӧm test for nicotine dependence (FTND) [39].

#### Follow-up measures

All participants will complete 1-, 3-, 6-, and 12-month follow-up assessments over the phone or online using the Tencent Questionnaire platform at participants’ convenience. Three non-participating staff will conduct the telephone follow-ups. The follow-up assessment information includes smoking status, quitting attempts, acceptability and satisfaction of the cessation services, and the usage of smoking cessation services, etc. A scale of 0 to 10 (0: not at all; 10: very high) for acceptability and satisfaction with each service will be evaluated. Participants who report quitting smoking or reducing their consumption of cigarettes will fill out the Minnesota Nicotine Withdrawal Scale (MNWS) [40] and the Brief- Questionnaire of Smoking Urges (QSU Brief) [40]. Adverse events and other unintended effects of interventions during the intervention period will be spontaneously reported. At 1-month follow-up, individuals with self-reported smoking abstinence for more than 7 days will be invited to participate in an exhaled carbon monoxide (CO) test for biochemical validation. To increase follow-up compliance, participants will receive 100 RMB (approximately US $ 14.95) in the form of the WeChat red packet (Hongbao, which is similar to an electronic cash reward [41]). The full list of measurements is presented in Table 3.

**Table 3:**
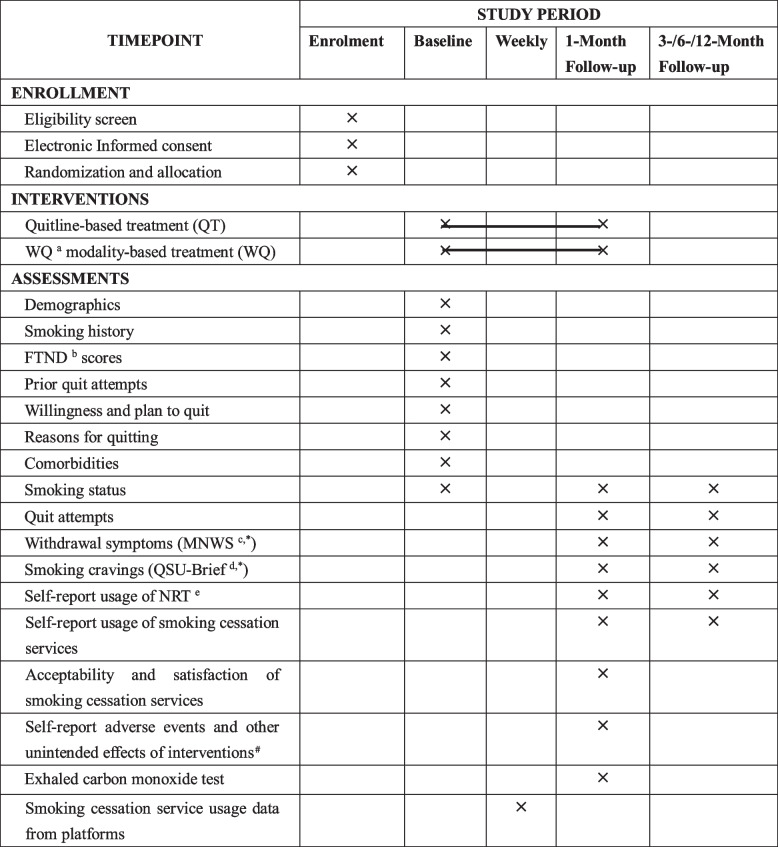
SPIRIT flow diagram of enrollment, interventions, and assessments

^a^WQ modality, “Way to Quit” modality

^b^FTND, Fagerstrӧm test for nicotine dependence

^c^MNWS, Minnesota Nicotine Withdrawal Scale

^d^QSU Brief, Brief-Questionnaire of Smoking Urges

^e^NRT, Nicotine replacement therapy

^*^Only for participants who have quit smoking or reduced the cigarette consumption

^#^Only for participants with self-reported smoking abstinence for more than 7 days

#### Service usage data

Service usage data will also be downloaded from the WeChat app and quitline platforms on a weekly basis during the intervention period. The QUIT WMP utilization metrics (registration, logins) will be extracted from host servers; participation in the QUIT WG (e.g., retention time and the number of messages sent) will be extracted. Telephone counsellors used a web-based tool to record the number, duration and content of calls.

#### Cost data

The cost of each service will be recorded through questionnaire, staff recording, and interview, including direct operating expenses (e.g., salary of staff members and materials used for the interactive activities and training of counselors), costs for hosting the quitline and WeChat-based services, technical support and recruitment costs (which consist of both advertising costs in web-based and offline media, as well as the costs of printing promotional material), and costs for promoting intervention delivery (e.g., rewards for participating in the interactive activities in the QUIT WG).

#### Outcomes

The primary outcome is biochemically validated 7-day point prevalence of abstinence (PPA) at the 1-month follow-up [42], defined as the proportion of smokers who self-report not even a puff of smoke in the past 7 days at the 1-month follow-up and are confirmed by a carbon monoxide level in expired air of less than 3 parts per million (ppm) [43].

Secondary outcomes include self-reported 7-day PPA [42] at the 1-, 3-, 6-, 12-month follow-up, self-reported continuous smoking abstinence rate (defined as the proportion of participants who are smoking less than 5 cigarettes [42]) from the 1-month to 12-month follow-up, self-reported quit attempts rate (defined as the proportion of participants who are continuously smoking but have tried to quit smoking for at least 24 h [44]) at the 1-, 3-, 6-, 12-month follow-up, acceptability and satisfaction scores of the services, incremental cost-effectiveness ratio (ICER) of the services.

### Sample size

The sample size calculation is based on the primary aim of comparing biologically verified 7-day PPA between the QT group and the WQ group at the 1-month follow-up. We used the following assumptions: (1) significance level of 0.05 for a two-sided test; (2) statistical power of 90%; (3) biologically verified 7-day PPA at the 1-month follow-up in the QT group is conservatively estimated as 20% based on the data from California Smokers’ Helpline [19]; (4) an increase in abstinence rate of 15% in the WQ group compared with the QT group; (5) a dropout rate of 20%. Considering the random block size (4 or 6), 460 eligible patients (230 for each group) will be required in this study.

### Data management

Data from all questionnaires and assessment scales will be collected electronically via the Tencent questionnaire platform. These data will be integrated with data on the use of smoking cessation services downloaded from the Quitline and WeChat platforms. Participants' cell phone numbers were used as unique identifiers.

All study files will be stored securely in password-protected computer files. Only the principal investigators of the study (SLC and LRL) will be given access to the cleaned full data sets. A data monitoring committee (DMC) has been established and composed of clinicians and statisticians independent of clinical trials, which will be responsible for monitoring study conduct, safety, effectiveness and adaptive design. It is independent from the sponsor and has no role in the design of this study and will not have any role during its execution, analyses, interpretation of the data, or decision to submit results.

### Statistical analysis plan

All statistical analyses be analyzed and blinded to the intervention assignment by a statistician using SPSS version 22.0 software (SPSS, Inc., Chicago, IL). Data will be presented as mean (standard deviations) for continuous variables with normal distribution; median (inter-quartile range) for continuous variables without normal distribution; and proportions for categorical variables. Baseline characteristics of participants in the intervention and control groups will be compared using Student’s t-test or non-parametric test for continuous variables and Pearson's chi-square or Fisher's exact test for categorical variables.

Multiple logistic regression will be used for between-group comparisons of the primary outcome variable-biochemically validated 7-day PPA at the 1-month follow-up and the secondary outcomes- self-reported 7-day PPA at the 1-, 3-, 6-, and 12-month follow-up and self-reported continuous smoking abstinence from 1-month to 12-month, with odds ratio (OR) and 95% confidence intervals (CI) to assess between-group effects. By intent-to-treat analysis (ITT), participants who are lost to follow-up or refuse to participate in the validation tests will be considered as continuing smokers with no reduction in cigarette consumption compared with baseline. Participants who do not use smoking cessation services after randomization will be excluded from the ITT analysis data set. In addition, we will conduct a per-protocol (PP) analysis of the primary outcome among the participants in the QT group who receive at least one telephone session and those in the WQ group who use the QUIT WMP for at least 20 days and send more than 10 short messages in the QUIT WG. For comparisons of secondary continuous outcomes with non-normal distribution assumptions, Mann–Whitney tests will be conducted. Subgroup analyses will assess the efficacy in participants with different demographics, baseline smoking characteristics, combined chronic diseases, and use of intervention variables (including compliance of using NRT gum, frequency and duration of using WeChat- or quitline-based cessation services) using multiple logistic regression. No interim analysis will be conducted. A two-sided *P* value of < 0.05 will be considered as statistically significant difference.

An economic evaluation will be conducted alongside this RCT following the approach of Drummond et al. [45] and in concordance with the Consolidated Health Economic Evaluation Reporting Standards statement [46]. The ICER will be calculated as follows: ICER = (C1 − C0) / (E1 − E0), where C refers to costs, E refers to effect, and the subscripts 1 and 0 refer to the WQ and QT groups, respectively. We will generate 2500 replicate samples by bootstrap and estimate the corresponding incremental costs and effects for each replicate sample, which are then plotted on a cost-effectiveness plane.

## Discussion

Currently, many mHealth-based interventions for smoking cessation are available, but they often have limited features [10]. Interventions based on SMS texting [14] or the web [47] frequently fail to deliver the recommended elements of behavioral treatments due to non-tailored content and lack of interactivity. The smartphone apps combine elements of texting and the web to create tailored and more interactive interventions [25]. The majority of them, however, failed to follow smoking cessation treatment guidelines and behavior change techniques, which led to poor quality interventions and lowered their effectiveness [48–51]. Moreover, their effectiveness was limited in real-world settings due to the high attrition and low utilization rates [52]. Social media could provide online guidance and increase the interactivity between smokers, but its effectiveness is still uncertain due to the low intensity of intervention [30–32]. In light of these above mentioned, the Surgeon General's 2020 report suggested integrating multiple treatment resources, as a potential strategy for increasing the reach and engagement of mHealth-based interventions for smoking cessation [10], while at the same time maintaining or improving their effectiveness.

Using the WQ modality, three mHealth-based interventions can be integrated into one platform, enabling information to be transferred between different services as well as mutual referrals, thereby increasing the reach of interventions and improving their effectiveness as a result of the cooperative effect [10]. Moreover, it was developed using the I-CET model, which incorporates several behavioral change theories (COM-B [20], EST [21], and TTM [22]), and provides evidence-based, comprehensive, stag-matched, and tailored smoking cessation interventions to fit the needs of the individual.

Through the QUIT WMP, smokers can develop skills to quit smoking, receive tailored tips, and obtain motivation to quit. It was developed based on guidelines for smoking cessation interventions [10, 29] and behavior change techniques [28], ensuring its efficacy and quality. Moreover, it could be accessed directly through the WeChat app without the need to download or install an application [41], which eliminates some limitations associated with smartphone apps. A pilot RCT has preliminarily found that WeChat mini-program might be effective in helping smokers quit smoking (biochemically verified 7-day PPA at 6 weeks in intervention group was 25%), but it was only self-help interventions, not combined with other interventions, such as online guidance by social media [53]. As part of the WQ modality, WeChat mini-program was integrated with other interventions, which is expected to be more effective than using WeChat alone. Our cohort study conducted in 12 provinces and cities in western China has preliminarily indicated that the WQ modality was effective in assisting Chinese smokers in quitting (self-reported 7-day PPA at one-month was 41.8%) [18].

The QUIT WG could offer group interventions led by professionals on a one-to-many base [54]. Intensive comprehensive cessation treatment at the group level likely brings to bear many key intervention functions of BCW [20], such as education, persuasion, training, modelling and environmental restructuring, all of which have consistently led to high quit rates [54]. And the online manner could broaden the reach and availability of group interventions [10]. Moreover, the QUIT WG can provide smokers with real-time online cessation counseling, which increases their knowledge about cessation, boosts their motivation and self-efficacy, and imparts their skills to quit [20, 28]. More importantly, “Quitting Pioneer” in the group could provide an example for other smokers to aspire to or imitate, share their experiences in quitting, motivate other smokers to quit and encourage them to participate in interactive activities [33, 34, 36]. In addition to enhancing the credibility and adherence of interventions among smokers, these interventions will facilitate the creation of reciprocal, strong, and long-lasting social bonds that promote smoking cessation. As well, the group administrator could supervise and guide smokers in using the QUIT WMP, which would increase its usage and improve its effectiveness.

The primary outcome of this study is slightly different from that of other related studies. The Society for Research on Nicotine and Tobacco recommends that the effectiveness outcome at the end of the intervention should be the primary outcome for determining whether the treatment is effective [42]. In light of this, we choose the short-term (1-month) outcome as the primary outcome based the intervention period (four weeks) rather than the long-term (such as 6-months or 12-months) outcomes selected by other studies [55]. Nevertheless, most of other studies have reported 1-month outcome [55], and we will also collect the long-term (3-month, 6-month, and 12-month) outcomes as secondary outcomes. Thus, the results of this study will be comparable to those of other studies.

Limitations should be mentioned. Firstly, the intervention period in this study has a shorter intervention period (four weeks) than other related studies (more than three months) [55]. This is primarily due to the relatively low engagement of mHealth-based approaches over a prolonged period, and the highest activity often occurs in the first month [56]. In the future, we will extend the intervention period once the effectiveness of this short-term intervention modality has been confirmed, with the intention of improving the long-term effectiveness of the WQ modality. Secondly, this study is an open label study, which may lead to information bias. In order to compensate for this type of bias, a variable random block length can be used instead of a fixed length, such as four or six. Furthermore, the staff performing the telephone follow-ups and statistical analysis will be blinded, and the participants’ self-reported abstinence will be biochemically validated by the exhaled CO test. Thirdly, this is a single-center study which could limit the generalizability of the results. Finally, all participants will be young to middle-aged, as elderly individuals have difficulty utilizing mobile devices skillfully [57]. Accordingly, the results should be extrapolated with caution to the elderly population.

It is anticipated that this trial, along with its findings will provide evidence for the WQ modality as an effective, acceptable, and affordable mHealth-based approach to smoking cessation. It will be of paramount significance to reduce health disparities as well as address the imbalance in smoking cessation services between regions and socioeconomic groups.

## Supplementary Information

**Additional file 1.** SPIRIT 2013 Checklist: Recommended items to address in a clinical trial protocol and related documents*.

**Additional file 2.** TheQuick Response code of the QUIT WeChat mini-program.

## Data Availability

Data about individual identified participants of this study will be available from the corresponding author on reasonable request after the main results of the study have been published.

## Abbreviations

Apps: Applications
BCW: Behavior change wheel
CI: Confidence intervals
CO: Carbon monoxide
COM-B: Capability, Opportunity, Motivation, and Behavior
DMC: Data monitoring committee
EST: Ecological Systems Theory
FTND: Fagerstrӧm test for nicotine dependence
GATS: Global Adult Tobacco Survey
ICER: Incremental cost-effectiveness ratio
I-CET model: Integrated COM-B/ Ecological systems/ Transtheoretical model
ITT: Intent-to-treat
mHealth: Mobile health
MNWS: Minnesota Nicotine Withdrawal Scale
NRT: Nicotine replacement therapy
OR: Odds ratio
PP: Per-protocol
PPA: Point prevalence of abstinence
ppm: Parts per million
QR: Quick Response
QSU Brief: Brief-Questionnaire of Smoking Urges
QT: Quitline-based treatment
RCT: Randomized controlled trial
SMS: Short message service
SPIRIT: Standardized protocol items recommendations for interventional trials
TTM: Transtheoretical Model
WMP: WeChat mini program
WG: WeChat group
WOA: WeChat official account
WQ modality: “Way to Quit” modality
