# Supplementary figures and images for: Evaluation of an innovative mHealth-based integrated modality for smoking cessation in Chinese smokers: protocol for a randomized controlled trial
Source: BMC Public Health. 2023 Mar 25;23:561. doi: 10.1186/s12889-023-15448-7 (PMC10038776; doi:10.1186/s12889-023-15448-7)

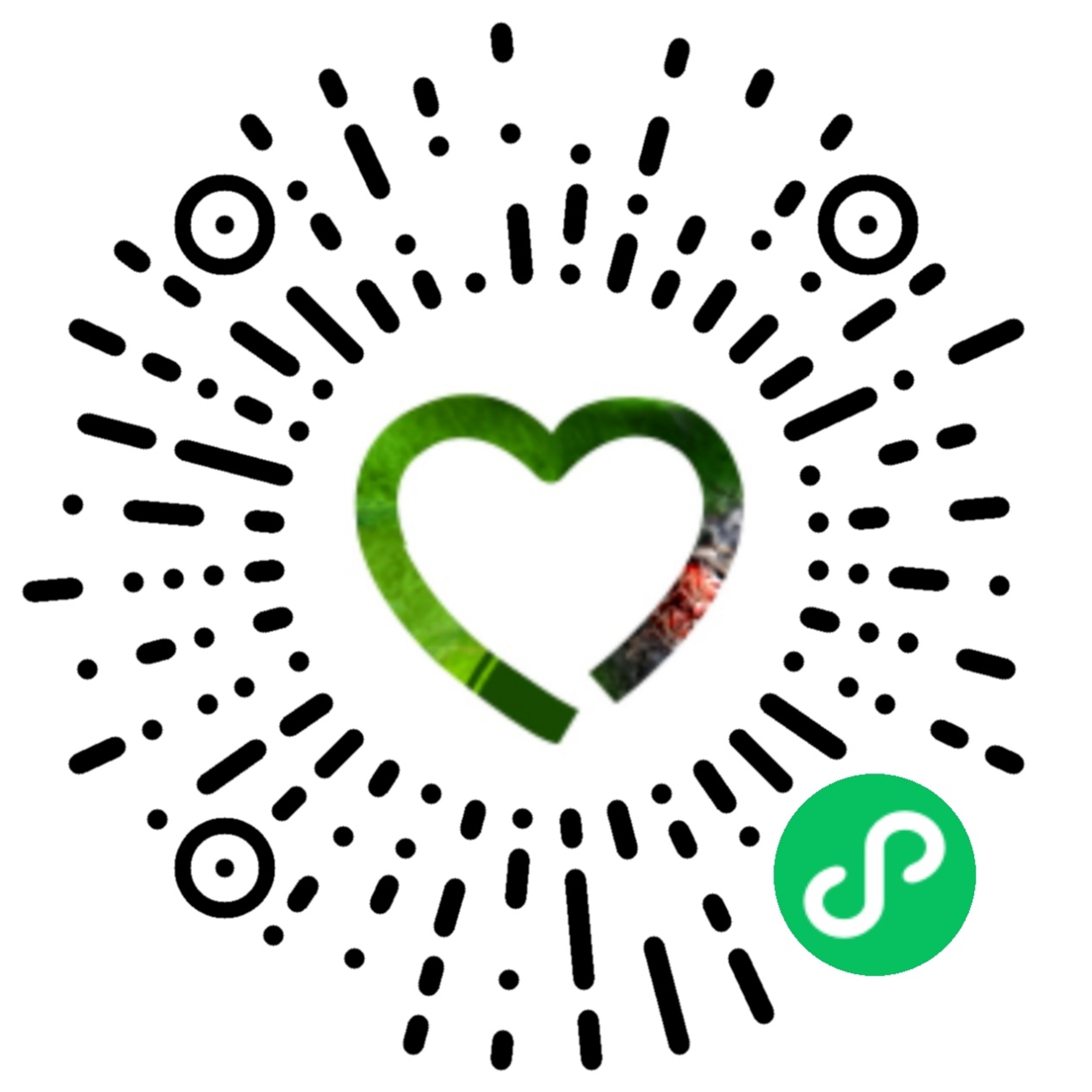


Additional 2. The Quick Response code of the QUIT WeChat mini-program

Supplement: Supplementary file 2 — Additional file 2. TheQuick Response code of the QUIT WeChat mini-program. [file 12889_2023_15448_MOESM2_ESM.docx]
